# Patient expectations, experiences and satisfaction with nintedanib and pirfenidone in idiopathic pulmonary fibrosis: a quantitative study

**DOI:** 10.1186/s12931-020-01458-1

**Published:** 2020-07-23

**Authors:** C. C. Moor, R. L. M. Mostard, J. C. Grutters, P. Bresser, J. G. J. V. Aerts, C. D. Dirksen, M. L. Kimman, M. S. Wijsenbeek

**Affiliations:** 1 Department of Respiratory Medicine, Erasmus Medical Center, Dr. Molewaterplein 40, 3015 GD Rotterdam, the Netherlands; 2 Department of Respiratory Medicine, Zuyderland Medical, Heerlen, the Netherlands; 3 Interstitial Lung Diseases Centre of Excellence, Department of Pulmonology, St Antonius Hospital, Nieuwegein, the Netherlands; 4 Division of Heart & Lungs, University Medical Center Utrecht, Utrecht, the Netherlands; 5 Department of Respiratory Medicine, Onze Lieve Vrouwe Gasthuis, Amsterdam, the Netherlands; 6 Department of Clinical Epidemiology and Medical Technology Assessment, Maastricht University Medical Centre, Maastricht, The Netherlands

**Keywords:** Idiopathic pulmonary fibrosis, Patient-reported outcomes, Patient satisfaction, Medication, Patient experiences

## Abstract

**Background:**

Two antifibrotic drugs, nintedanib and pirfenidone, are available for treatment of idiopathic pulmonary fibrosis (IPF). Although efficacy and adverse events have been well studied, little is known about patient experiences with these drugs. We aimed to systematically and quantitatively evaluate patient expectations, experiences, and satisfaction with nintedanib and pirfenidone. Furthermore, we assessed which factors were associated with overall patient satisfaction with medication.

**Methods:**

Outpatients with IPF prospectively completed the Patient Experiences and Satisfaction with Medication (PESaM) questionnaire before start, and after three and 6 months of antifibrotic treatment, as part of a randomized eHealth trial (NCT03420235). The PESaM questionnaire consists of an expectation module, a validated generic module evaluating patient experiences and satisfaction concerning the effectiveness, side-effects, and ease of use of a medication, and a disease-specific module about IPF. Satisfaction was scored on a scale from − 5 (very dissatisfied) to + 5 (very satisfied).

**Results:**

In total, 90 patients were included, of whom 43% used nintedanib and 57% pirfenidone. After 6 months, the mean overall score for satisfaction with medication was 2.1 (SD 1.9). No differences were found in experiences and satisfaction with medication, and the number and severity of side-effects between nintedanib and pirfenidone. Perceived effectiveness of medication was rated as significantly more important than side-effects and ease of use (*p* = 0.001). Expectations of patients regarding effectiveness were higher than experiences after 6 months. Self-reported experience with effectiveness was the main factor associated with overall medication satisfaction.

**Conclusions:**

Patient experiences and satisfaction with antifibrotic treatment were fairly positive, and similar for nintedanib and pirfenidone. Systematic evaluation of patient expectations, experiences, and satisfaction with medication could enhance shared-decision making and guide drug treatment decisions in the future.

**Trial registration:**

NCT03420235.

## Background

Idiopathic pulmonary fibrosis (IPF) is a rare, progressive interstitial lung disease with a poor prognosis [1]. Two antifibrotic drugs, nintedanib and pirfenidone, are available for treatment of IPF. These drugs slow down disease progression, may reduce the rate of acute exacerbations, and seem to prolong survival [2–4]. The decision to prescribe either nintedanib or pirfenidone is usually based on the specific side-effect profiles, comorbidities, co-medication, and a patient’s preferences [5, 6]. The prevalence of adverse events has been reported in randomized controlled trials and registries with real-world data. In these studies, around 20–30% of patients permanently discontinued antifibrotic treatment due to adverse events, such as diarrhea or photosensitivity [7, 8]. Although efficacy and adverse events have been well studied, little is known about patient experiences and satisfaction with antifibrotic medication. Earlier qualitative studies suggested that experiences and satisfaction with medication were relatively positive in most patients [9–11]. Patients with side-effects reported to have lower satisfaction levels and an impaired (health-related) quality of life ((HR)QOL) [10]. Studies in other chronic diseases showed that patient satisfaction with medication can influence health-related decisions and compliance with medication [12, 13]. Consequently, experiences and satisfaction with medication may also affect long-term treatment outcomes [12]. Moreover, expectations before start of treatment are associated with outcomes and satisfaction in other chronic conditions [14]. Gaining better insights in patient expectations before start of treatment could help to optimize expectation management and aid shared-decision making [14].

In recent years, it has been increasingly acknowledged that the patient perspective should play a central role in treatment decisions in IPF [6, 15]. Shared-decision making is only possible if patients’ needs, expectations, experiences, and preferences are regularly (re) assessed during the disease course. To allow for structured collection and evaluation of patient expectations and experiences with pharmacological treatment, the patient experiences and satisfaction with medication (PESaM) questionnaire has recently been developed and validated in IPF [11, 15].

In this study, we aimed to evaluate patient expectations, experiences, and satisfaction with antifibrotic treatment using the PESaM questionnaire. Furthermore, we compared PESaM scores between patients using nintedanib and pirfenidone, evaluated the relationship between (health-related) quality of life and satisfaction with medication, and assessed which factors were associated with overall medication satisfaction.

## Methods

### Study design and participants

Outpatients with IPF prospectively completed the PESaM questionnaire before start, and after three and 6 months of antifibrotic treatment, as part of a multi-center randomized home monitoring trial at four sites in the Netherlands [16]. The trial is registered on www.clinicaltrials.gov (NCT03420235). Eligible to participate were adults (> 18 years) with a diagnosis of IPF confirmed in a multidisciplinary team meeting, according to the ATS/ERS/JRS/ALAT guideline, who were about to start on antifibrotic treatment [1]. This study was approved by the institutional review board of all participating centers. All patients provided written informed consent prior to study entry. Patients also completed the King’s Brief Interstitial Lung Disease questionnaire (K-BILD), EQ-5D-5L, Hospital Anxiety and Depression Scale (HADS), and a visual analogue scale (VAS) on symptoms [17–19]. All questionnaires were completed online in a secured application on a tablet, before the doctor’s visit. When the use of antifibrotic medication was discontinued during the study, follow-up PESaM questionnaires were not completed. Forced vital capacity (FVC) and diffusion capacity of the lung for carbon monoxide (DLCO) were performed at all hospital visits.

### Outcome measures

The PESaM questionnaire evaluates patient expectations (only before start of drug treatment), experiences, and satisfaction regarding effectiveness, side-effects, and ease of use of medication, and its impact on a patient’s health and daily activities. The PESaM consists of three modules; an expectation module, a generic module applicable to any medication, and a disease-specific module, especially for IPF. The expectation module consists of 11 questions reported on a 5-point Likert scale from 0 to 4. Questions concern the expected effectiveness, bothersomeness of side-effects, and ease of use of a medication; higher scores indicate more positive expectations. The generic module includes 18 items in three domains (effectiveness, side-effects, ease of use). Patient experiences are scored similar to expectations on a Likert scale from 0 to 4, with higher scores representing more positive experiences. Satisfaction is scored on a horizontal thermometer with scores ranging from − 5 (very dissatisfied) to 5 (very satisfied). Finally, patients scored how important they considered effectiveness, side-effects, and ease of use of their medication (0 = not important at all, 4 = very important). The disease-specific module contains 10 items, and evaluates experiences with disease-specific symptoms and side-effects. Information on generic and disease-specific topics in the PESaM questionnaire can be found in supplementary Table 1. More detailed information regarding the development, scoring and validation of the PESaM questionnaire has previously been described [11, 15].

The K-BILD consists of 15 items in four different domains (total score, psychological domain, breathlessness and activities, and chest domain). Scores range from 0 to 100 with higher scores indicating a better HRQOL [17]. The EQ-5D-5L comprises 5 questions and a visual analogue scale on general wellbeing from 0 to 100; higher scores represent a better QOL [18]. The HADS is divided in a 7-item anxiety and 7-item depression scale, with scores ranging from 0 to 21. A score of 8 or higher indicates anxiety or depressive symptoms [19]. Symptoms (cough, dyspnea, and general complaints) were scored on a VAS from 0 to 10.

### Statistical analysis

Analyses were conducted in patients who completed the PESaM questionnaire at ≥1 time point. Differences between nintedanib and pirfenidone were analyzed with independent students t-tests at three and 6 months. Paired students t-tests were used to analyze differences between scores at three and six months in the overall group. Expectations before start of treatment and experiences after six months were analyzed on item level with Wilcoxon Signed Ranks tests. Correlations between (HR)QOL and satisfaction at six months were analyzed with Pearson correlation coefficients. Differences in patient-reported importance of effectiveness, side-effects and ease of use were analyzed using repeated measures ANOVA with post-hoc tests (Bonferroni correction). A linear regression model was used to analyze factors predictive for satisfaction with medication after six months. Variables included in the univariable analysis were age, gender, and patient expectations at baseline, lung function, type of antifibrotic drug, symptoms, HADS scores, and self-reported experiences with effectiveness, side-effects, ease of use, and severity of side-effects at six months, and change in FVC and DLCO % of predicted over time. Variables with a *p*-value < 0.10 on the univariable analysis were included in the multivariable analysis (enter method). A p-value < 0.05 was considered statistically significant. All statistical analyses were performed in SPSS Statistics version 25.

## Results

A total of 90 patients were included, of whom 43% used nintedanib and 57% pirfenidone. Of these patients, 83 completed the PESaM at baseline, 83 after three months, and 78 after six months. Baseline characteristics were comparable between both groups, except for FVC (Table 1). During the study, two patients died, five patients discontinued antifibrotic treatment due to side-effects, and seven patients switched medication.

**Table 1:** Baseline characteristics of study patients (*n* = 90)

	Nintedanib (***n*** = 39)	Pirfenidone (***n*** = 51)	***p***-value
Age, years	70 (7)	72 (7)	0.22
Male sex – no. (%)	35 (90)	47 (92)	0.72
FVC % predicted	86 (16)	76 (16)	0.01
FVC (L)	3.4 (0.9)	2.9 (0.6)	0.01
DLCO % predicted	48 (14)	49 (14)	0.69
K-BILD total score	56.5 (9)	56.7 (10)	0.89
EQ 5D-Index value	0.78 (0.15)	0.77 (0.19)	0.78
EQ5D-VAS scale	65 (24)	63 (23)	0.66
VAS – cough*	4.8 (2.6)	4.6 (2.5)	0.66
VAS – dyspnea*	5.1 (2.3)	5.6 (2.4)	0.43
VAS – general complaints**	5.6 (2.4)	5.5 (2.4)	0.75
HADS – depression score	3.0 (3.1)	3.9 (3.5)	0.21
HADS – anxiety score	4.7 (2.3)	4.6 (2.3)	0.94

Data are presented as mean (SD). *FVC* forced vital capacity, *DLCO* diffusion capacity of the lung for carbon monoxide, *K-BILD* King’s Brief Interstitial Lung Disease questionnaire, *VAS* visual analogue scale, HADS Hospital Anxiety and Depression Scale. * a higher score represent worse symptoms, ** a higher score represents fewer symptoms

### Expectations, experiences, and satisfaction in the overall group

In the overall group, there were no significant differences in experiences and satisfaction after three and after six months (Table 2). Expectations of patients regarding effectiveness (mean score 2.8, SD 0.8) and side-effects (mean score 2.5, SD 0.8) were positive. At six months, the mean score for experiences with effectiveness (score 2.0, SD 1.0) was lower than the expectation score (difference 0.8, *p* = 0.001). Experiences with side-effects (mean score 2.9, SD 1.1) were comparable with expectations (difference 0.4, *p* = 0.07). Many patients chose the answer option “don’t know” for questions in the expectation module. Hence, expectations and experiences could only be compared in a relatively small number of patients (*n* = 26 for effectiveness, *n* = 39 for side-effects). Expectations, experiences and satisfaction were similar across treatment centers.

**Table 2:** Patient experiences and satisfaction with antifibrotic treatment after three and six months in the overall group (*n* = 75)

	Month 3	Month 6	Difference (95% CI)	*p*-value
Satisfaction with effectiveness	1.6 (1.6)	1.6 (1.8)	0.0 (− 0.3–0.5)	0.70
Satisfaction with side-effects	1.8 (2.0)	1.6 (2.1)	0.2 (− 0.4–0.6)	0.57
Satisfaction with ease of use	2.9 (1.6)	2.7 (1.7)	0.2 (− 0.1–0.6)	0.18
Overall satisfaction with medication	2.1 (1.8)	2.1 (1.9)	0.0 (−0.4–0.5)	0.90
Experiences with effectiveness	2.0 (0.9)	2.0 (1.1)	0.0 (−0.4–0.5)	0.84
Experiences with side-effects	3.1 (1.1)	2.9 (1.2)	0.2 (−0.3–0.5)	0.44
Experiences with ease of use	3.9 (0.5)	3.8 (0.5)	0.1 (−0.1–0.2)	0.38
Number of reported side-effects per patient	6.4 (4.2)	5.8 (4.7)	0.6 (−0.2–1.4)	0.14
Severity score side-effects	9.5 (11.1)	8.7 (9.3)	0.8 (−1.6–3.2)	0.51

Data are presented as mean (SD). Experiences are scored on a scale from 0 to 4; a higher score corresponds with more positive experiences. Satisfaction is scored on a scale from − 5 to 5

After six months, patients rated how important they considered the effectiveness, side-effects, and ease of use of their antifibrotic medication. Effectiveness (mean score 3.5, SD 0.8) was rated as significantly more important than side-effects (mean score 2.2, SD 1.3), and ease of use (mean score 1.8, SD 1.3), *p* = 0.001. Self-reported adherence with antifibrotic medication after six months was high: 88% of patients reported 100% adherence, 10% reported that they missed one or a few pills, and 2% reported that they often skipped their medication in the past four weeks.

### Comparison between nintedanib and pirfenidone

Expectations before start of treatment were similar for nintedanib and pirfenidone. No differences were found in experiences and satisfaction between nintedanib and pirfenidone after three and six months of antifibrotic treatment (Table 3). Moreover, the reported number and severity of side-effects were similar in patients using nintedanib and pirfenidone. For nintedanib, the most frequently reported side-effects were diarrhea (70.3%), fatigue (56.8%), and abdominal pain (45.9%). For pirfenidone, the most frequently reported side-effects were fatigue (68.3%), skin-related events (58.5%), and decreased appetite (53.7%).

**Table 3:** Experiences and satisfaction with nintedanib and pirfenidone after three and six months of antifibrotic treatment

	Month 3				Month 6			
	Nintedanib (***n*** = 37)	Pirfenidone (***n*** = 46)	Difference (95% CI)	***p***-value	Nintedanib (n = 37)	Pirfenidone (***n*** = 41)	Difference (95% CI)	***p***-value
Satisfaction with effectiveness	1.7 (1.6)	1.5 (1.6)	0.2 (− 0.5–0.9)	0.58	1.4 (1.7)	1.7 (1.9)	0.3 (− 1.1–0.6)	0.54
Satisfaction with side-effects	1.7 (2.0)	1.8 (2.2)	0.1 (− 1–0.9)	0.9	1.3 (1.9)	1.8 (2.2)	0.4 (− 1.4–0.5)	0.37
Satisfaction with ease of use	3.2 (1.6)	2.6 (1.6)	0.6 (− 0.1–1.3)	0.08	2.9 (1.4)	2.5 (1.9)	0.4 (−0.4–1.1)	0.3
Overall satisfaction with medication	2.2 (1.7)	1.9 (1.8)	0.2 (−0.5–1)	0.53	1.9 (1.7)	2.2 (2.0)	0.2 (−1.1–0.6)	0.58
Experiences with effectiveness	1.7 (1.0)	1.7 (0.9)	0.09 (−0.7–0.5)	0.75	2 (1.1)	1.8 (1.0)	0.2 (− 0.6–1)	0.55
Experiences with side-effects	3.1 (1.2)	2.9 (1.2)	0.3 (−0.3–0.8)	0.41	2.8 (1.2)	3.1 (1.1)	0.3 (−0.8–0.2)	0.25
Experiences with ease of use	3.9 (0.3)	3.8 (0.5)	0.1 (−0.05–0.3)	0.14	3.9 (0.3)	03.7 (0.6)	0.1 (−0.07–0.4)	0.18
Number of reported side-effects per patient	6.0 (3.5)	6.6 (4.6)	0.6 (−2.4–1.3)	0.55	5.7 (4.2)	5.7 (5.1)	0.05 (−2–2.1)	0.96
Severity score side-effects	9.9 (13.2)	9.8 (9.3)	0.1 (−4.8–5.1)	0.96	8.9 (9.5)	8.3 (9.0)	0.6 (−3.3–4.5)	0.75

Data are presented as mean (SD). Experiences are scored on a scale from 0 to 4; a higher score corresponds with more positive experiences. Satisfaction is scored on a scale from − 5 to 5

### Relation between satisfaction with medication and (HR)QOL

Total scores of K-BILD and EQ-5D-5L did not change over six months. Overall, moderate correlations were found between (HR)QOL and satisfaction with medication at six months. The total K-BILD score was moderately correlated with satisfaction regarding effectiveness (*r* = 0.57, *p* = < 0.001), side-effects (*r* = 0.51, *p* < 0.001), ease of use (*r* = 0.42, *p* < 0.001), and overall medication satisfaction (*r* = 0.46, *p* < 0.001). The EQ-5D-VAS score also showed a moderate correlation with overall satisfaction (*r* = 0.59, *p* < 0.001), satisfaction with side-effects (*r* = 0.48, *p* < 0.001), ease of use (*r* = 0.53, *p* < 0.001) and effectiveness (*r* = 0.58, *p* < 0.001).

### Factors associated with overall satisfaction with medication

Experiences with effectiveness, side-effects and ease of use, severity of side-effects, change in FVC% predicted over time, anxiety and depression scores, cough, dyspnea, and general complaints were significantly associated with overall satisfaction after six months on univariable linear regression analysis (Table 4). Patients’ expectations before start of treatment, lung function parameters, DLCO change over time, the type of antifibrotic drug, age and gender were not associated with patient satisfaction on univariable analysis. Change in FVC% predicted over time was included in the multivariable analysis, as the *p*-value was < 0.10. Because of the strong relation between cough and dyspnea, we chose to include general complaints in the multivariable model, as a proxy for cough and dyspnea. In the multivariable model, only experiences with effectiveness was significantly associated with satisfaction with medication (Table 4).

**Table 4:** Univariable and multivariable linear regression analyses of factors associated with overall satisfaction with medication (*n* = 78)

	Univariable analysis		Multivariable analysis	
	B (95% CI)	***p***-value	B (95% CI)	***p***-value
Age	0.019 (− 0.04;0.08)	0.55	–	–
Gender	−0.97 (−2.6;0.6)	0.25	–	–
Expectations effectiveness	0.54 (−0.15;1.2)	0.12	–	–
Expectations side-effects	−0.10 (− 0.85;0.65)	0.79	–	–
FVC % predicted	0.004 (−0.02;0.03)	0.76	–	–
DLCO % predicted	0.008 (−0.02;0.04)	0.60		
Change in FVC % predicted*	0.05 (−0.005;0.01)	0.079	0.026 (−0.05;0.10)	0.64
Change in DLCO % predicted*	−0.04 (− 0.08;0.01)	0.11	–	–
Antifibrotic drug	0.24 (−0.62;1.1)	0.58	–	–
VAS - cough	−0.23 (− 0.39;-0.07)	0.005		
VAS – dyspnea	−0.41 (− 0.56;-0.26)	< 0.001		
VAS - general complaints	0.36 (0.15;0.57)	0.001	0.04 (−0.34;0.42)	0.84
HADS – anxiety score	−0.30 (− 0.47;-0.14)	0.001	− 0.23 (− 0.63;0.17)	0.24
HADS – depression score	− 0.24 (− 0.35;-0.13)	< 0.001	−0.01 (− 0.29;0.28)	0.95
Experiences effectiveness	1.2 (0.6;1.75)	< 0.001	0.95 (0.22;1.67)	0.01
Experiences side-effects	−0.53 (− 0.87;-0.18)	0.003	0.07 (− 0.67;0.82)	0.84
Experiences ease of use	−0.99 (−1.8;-0.17)	0.019	−0.34 (−1.99;1.31)	0.68
Severity score side-effects	−0.07 (− 0.11;− 0.03)	0.001	-0.03 (− 0.11;0.052)	0.49

*FVC* forced vital capacity, *DLCO* diffusion capacity of the lung for carbon monoxide, *HADS* hospital anxiety and depression scale, *VAS* visual analogue scale, *change in FVC and DLCO between baseline and six months

## Discussion

In this study, we evaluated patient expectations, experiences and satisfaction with antifibrotic medication in patients with IPF, as this could aid future shared-decision making. To our knowledge, this was the first study in IPF which systematically assessed patient experiences with medication, using the validated PESaM questionnaire [15]. Patient experiences and satisfaction after three and six months of antifibrotic treatment were fairly positive, and similar in nintedanib and pirfenidone. Satisfaction with medication had a moderate positive correlation with (HR)QOL. Self-reported experiences with the effectiveness of the medication (e.g. positive impact on physical health, daily activities) was the main factor associated with overall medication satisfaction. Anxiety, depression, symptom scores, FVC change over time, experiences with side-effects, and perceived severity of side-effects were not associated with overall medication satisfaction after six months on multivariable analysis.

Interestingly, patients considered effectiveness of their medication more important than side-effects and ease of use. This is in line with findings from a recent study, which found that IPF patients were more concerned about slowing down disease progression than about side-effects. In contrast, almost a quarter of the surveyed healthcare providers in that study considered side-effects more important than the risk of disease progression [9]. Our results highlight the importance of shared-decision making, taking into account patients’ expectations, experiences and preferences in all treatment decisions, as patients’ opinions and considerations may be different than healthcare providers assume. In some settings, patients with a FVC above 80% do not receive antifibrotic treatment, either due to reimbursement restrictions or perceived barriers of doctors about early treatment [9]. In the current study, the mean FVC in the nintedanib group was 86%. Importantly, patient experiences and satisfaction with antifibrotic medication were also positive in this patient group with relatively high FVC, again underlining that from the patients’ perspective there is no rationale to delay treatment until lung function has declined below a FVC of 80%.

Overall, expectations regarding effectiveness were slightly higher than consecutive experiences, which emphasizes the need for realistic patient education and expectation management during the disease course [14]. Expectations and consecutive experiences with side-effects were comparable. There were no differences across participating centers. Expectation management may be complex in IPF, due to the heterogeneous disease course and the fact that antifibrotic medication does not halt or reverse lung function decline. Hence, it may be difficult for patients and their healthcare providers to judge the effectiveness of these drugs. This was reflected in the fact that a substantial number of patients answered that they did not know what to expect, and were uncertain whether treatment was effective. Nevertheless, the perceived effectiveness of medication was the only factor which was associated with overall medication satisfaction in a multivariable model. Patients’ beliefs and opinions regarding the effectiveness of their medication are factors known to impact medication adherence in other diseases [20]. In the present study, (self-reported) adherence with medication was very high. Thus, it could be speculated that uncertainty about effectiveness had no major influence on adherence. However, the design of the current study did not allow us to formally address this issue. Moreover, only a small minority of patient discontinued treatment (5.5%). The high adherence and low discontinuation rate could possibly be due to the relative short study duration. Longer prospective studies are needed to assess whether perceived effectiveness and satisfaction with medication affects long-term compliance and treatment outcomes [12, 15].

Previous studies have shown that nintedanib and pirfenidone have a similar effect on lung function decline. Furthermore, no significant differences in all-cause mortality and frequency of side-effects were found between the two drugs [2, 3, 5, 21]. However, these studies did not compare patient experiences and satisfaction between both antifibrotic drugs. Results of our study showed that self-reported experiences and satisfaction were similar in patients using nintedanib and pirfenidone. One could have speculated that the perceived ease of use would be different, because of the differences in dosing schedule. However, experiences and satisfaction with ease of use were very high in both groups, showing that patients considered it relatively easy to integrate the use of antifibrotic medication in their daily life. Although the side-effect profile was different for both drugs (e.g. more diarrhea in patients using nintedanib, more skin problems in patients using pirfenidone), the number and perceived severity of side-effects were similar. Use of structured tools like PESaM could facilitate insights into expectations, experiences and satisfaction in individual patients, and thereby guide decisions on treatment choices and adjustments throughout the disease course.

A strength of this study was that data were prospectively collected in a multi-center population of newly treated patients. Due to this multi-center design with multiple healthcare providers, potential differences in patient education about medication have been taken into account. This study has also limitations. The PESaM questionnaire has been developed and validated in IPF, but no minimal important difference and responsiveness has been established yet. Thus, further studies are needed to better explore clinical relevance of differences between patient expectations and experiences.

## Conclusions

Patient experiences and satisfaction with medication after three and six months of antifibrotic treatment were relatively positive, and comparable between nintedanib and pirfenidone. Patient expectations before start of treatment were high, emphasizing the need for realistic expectation management. Perceived effectiveness of medication was associated with overall medication satisfaction. The PESaM questionnaire is a novel, simple tool to evaluate patient satisfaction and experiences with medication, and can be used both in clinical trials and in daily practice. We believe that systematic evaluation of patient experiences could enhance shared-decision making and guide treatment decisions in the future.

## Supplementary information

**Additional file 1 Supplementary Table 1**. Overview of topics included in PESaM questionnaires in different modules

## Data Availability

The datasets used and/or analysed during the current study are available from the corresponding author on reasonable request.

## Abbreviations

IPF: Idiopathic pulmonary fibrosis
PESaM: Patient experiences and satisfaction with medication
HRQOL: Health-related quality of life
K-BILD: King’s brief interstitial lung disease questionnaire
HADS: Hospital anxiety and depression scale
VAS: Visual analogue scale
FVC: Forced vital capacity
DLCO: Diffusion capacity of the lung for carbon monoxide
