# Supplementary material for: Patient expectations, experiences and satisfaction with nintedanib and pirfenidone in idiopathic pulmonary fibrosis: a quantitative study
Source: Respir Res. 2020 Jul 23;21:196. doi: 10.1186/s12931-020-01458-1 (PMC7376884; doi:10.1186/s12931-020-01458-1)
Supplement: Supplementary file 1 — Additional file 1 Supplementary Table 1. Overview of topics included in PESaM questionnaires in different modules [file 12931_2020_1458_MOESM1_ESM.docx]

| **Supplementary table 1. Overview of topics included in PESaM questionnaires in different modules** | | | |
| --- | --- | --- | --- |
| **Domain** | **PESaM-module** | | |
|  | Disease-specific (IPF) | Generic experiences | Generic expectations |
| Effectiveness | - Coughing - Shortness of breath - Fatigue - Disease stabilisation | - Perceived effectiveness - Impact on physical health - Impact on feelings and emotions - Impact on social and daily activities - Satisfaction | - Expected effectiveness - Impact on physical health - Impact on feelings and emotions - Impact on social and daily activities |
| Side-effects | - Headache   - Insomnia - Fatigue - Dizziness - Weight loss - Decreased appetite - Photosensitivity - Other skin problems - Diarrhoea - Nausea - Vomiting - Flatulence - Stomach pain - Abdominal pain - Coughing - Other - Bothersomeness of each experienced side-effect - Non-adherence due to side-effects | - Bothersomeness experienced side-effects (any) - Impact on physical health - Impact on feelings and emotions - Impact on social and daily activities - Satisfaction | - Bothersomeness side-effects (any) - Impact on physical health - Impact on feelings and emotions - Impact on social and daily activities |
| Ease of use | - Intake capsules - Timing - Non-adherence due to inconvenience | - Administration mode - Time schedule or frequency - Incorporate into daily life - Satisfaction | - Administration mode - Time schedule or frequency - Incorporate into daily life |
| Other | N/A | - Overall satisfaction - Importance of effectiveness vs side-effects vs ease of use | N/A |
